# Perioperative factors associated with survival following surgery for pancreatic cancer – a nationwide analysis of 473 cases from Denmark.

**DOI:** 10.1186/s12893-024-02369-4

**Published:** 2024-03-02

**Authors:** Laura Marr Spore, Emilie Even Dencker, Eske Aasvang Kvanner, Carsten Palnaes Hansen, Stefan Kobbelgaard Burgdorf, Paul Suno Krohn, Sophie Louise Gisela Kollbeck, Jan Henrik Storkholm, Martin Sillesen

**Affiliations:** 1 Department of Organ Surgery and Transplantation, Copenhagen University Hospital, Rigshospitalet, Denmark; 2 Department of Anesthesia, Copenhagen University Hospital, Rigshospitalet, Denmark; 3 Institute of Clinical Medicine, University of Copenhagen Medical School, Copenhagen, Denmark; 4 Department of Surgery, Imperial College NHS Trust, Hammersmith Hospital, London, UK

**Keywords:** Pancreas, Cancer, Surgery, Transfusions, Anesthesia, Outcomes

## Abstract

**Background:**

Pancreatic ductal adenocarcinoma (PDAC) remains one of the most lethal cancers worldwide, with an overall 5-year survival rate of only 5%. The effect of perioperative treatment factors including duration of surgery, blood transfusions as well as choice of anesthesia and analgesia techniques on overall survival (OS) following pancreatic resections for PDAC, is currently not well known. We hypothesized that these perioperative factors might be associated with OS after pancreatic resections for PDAC.

**Methods:**

This is a retrospective study from a nationwide cohort of patients who underwent surgery for PDAC in Denmark from 2011 to 2020. Kaplan-Meier 1, 2 and 5-year survival estimates were 73%, 49% and 22%, respectively. Data were obtained by joining the national Danish Pancreatic Cancer Database (DPCD) and the Danish Anaesthesia Database (DAD). Associations between the primary endpoint (OS) and perioperative factors including duration of surgery, type of anesthesia (intravenous, inhalation or mixed), use of epidural analgesia and perioperative blood transfusions were assessed using Hazard Ratios (HRs). These were calculated by Cox regression, controlling for relevant confounders identified through an assessment of the current literature. These included demographics, comorbidities, perioperative information, pre and postoperative chemotherapy, tumor staging and free resection margins.

**Results:**

Overall, data from 473 resected PDAC patients were available. Multivariate Cox regression indicated that perioperative blood transfusions were associated with shorter OS (HR 2.53, *p* = 0.005), with survival estimates of 8.8% in transfused vs. 28.0% in non-transfused patients at 72 months after surgery. No statistically significant associations were identified for the duration of surgery or anesthesia/analgesia techniques.

**Conclusion:**

In this study, the use of perioperative blood transfusions was associated with shorter OS.

**Supplementary Information:**

The online version contains supplementary material available at 10.1186/s12893-024-02369-4.

## Introduction

Despite treatment advances, pancreatic ductal adenocarcinoma (PDAC) remains one of the most lethal cancer diseases, currently being the fourth leading cause of cancer-related deaths in the United States and Europe [1, 2]. The incidence has been steadily increasing, with projections indicating that PDAC could be the second leading cause of cancer-related death by 2035 [3]. Currently, surgical resection of the tumor remains the only curative option.

However, with a reported 3-year survival rate of 20–34% for successfully resected tumors [1] and an overall 5-year survival rate of 5% [4] it is clear that current treatment strategies are ineffective in addressing the increasing burden of PDAC.

Major research efforts have been invested in optimizing the oncological treatments, although with limited success so far. The addition of FOLFIRINOX adjuvant regimens have improved outcomes [5], but these are still highly dependent on the tumor stage at diagnosis. For successfully resected patients, current treatment regimens provide only a median overall survival of 54 months [6]. For patients with metastatic disease, this treatment may only provide a 5-month increase in median overall survival (OS) [5].

These findings highlight the limited progress seen in oncological treatment of PDAC in the past decades and emphasize the need to identify fields of potential treatment improvement outside of the oncological setting. Such areas could include optimization of the surgical treatment courses, including the perioperative phase.

Although the association between tumor size, locoregional metastases and unfavorable outcome is well established, studies have shown that perioperative factors may be of importance in promoting metastatic spread and thus early recurrence after otherwise successful tumor resections. Factors such as blood transfusions [7, 8], choice of anesthetic agent [9], regional anesthetics [10] and the surgical stress response [11] have all been assessed for potential associations with early recurrence with mixed reported results. Thus, knowledge of how these factors affect OS after pancreatic resections could potentially help to optimize perioperative treatment strategies with the aim of increasing OS.

The aim of this study was to identify perioperative factors associated with OS in a Danish PDAC cohort. We hypothesize that perioperative factors defined as perioperative blood transfusions, duration of surgery, choice of anesthetic method and use of epidural analgesia, may be associated with OS after PDAC surgery.

## Methods

This was a nationwide retrospective cohort study, including data from four institutions performing surgical treatment for PDAC. All surgical PDAC treatment in Denmark is performed at one of these four centers, with the volume of procedures varying from 20 to 250 per year between the centers. Data on overall results including key performance indicators such as number of planned vs. performed procedures (exploratory laparotomy rates), rate of R0 resections as well as 30-day and 1-year survival between the centers are continuously registered in the Danish Pancreatic Cancer Database (DPCD). Quality standards for these 4 centers require that > 80% of planned pancreatic resections to be performed are carried out (< 20% exploratory laparotomies due to unresectable tumors), resection rate > 95% R/R1, 30-day survival > 95%, and 1-year survival > 70%. All centers included in this study currently meet these quality standards.

Data were extracted from two national Danish registries, the DPCD and the Danish Anesthesia Database (DAD). Data from the DPCD included patients undergoing Pancreaticoduodenectomy (PD), total Pancreatectomies (TP) or Distal Pancreatectomies (DP) from May 1st, 2011, to December 31st, 2020. All included procedures were open procedures. Staging was performed according to the with the American Joint Committee on Cancer 8th edition criteria. PDAC diagnoses were histologically confirmed.

The DAD contains information on all anesthesia procedures in Denmark, including information on anesthesia techniques and perioperative transfusions.

The study was approved by the DPCD board of governors and the Danish Capital Region Data Protection authority with approval number: #P-2020-180. In accordance with Danish Law, informed consent, and approval by and ethics committee was not required due to the retrospective nature of this study. Specifically, the study adhered to national legislation as stipulated in the Danish Data Protection Law of May 2018, amendment 1509 § 10, Sects. 1 and 2. The study was carried out in accordance with the “Strengthening the reporting of observational studies in epidemiology” (STROBE) guidelines [12].

The associations between OS and one continuous (duration of surgery in minutes) as well as three discrete variables were investigated. Discrete variables were perioperative blood transfusions (packed red blood cells, PRBC. Information on transfusion of plasma and platelet products was not available), choice of anesthesia technique (total intravenous anesthesia, TIVA, or mixed TIVA/inhalation anesthesia) and use of epidural analgesia. Information on the volume of PRBC was not available from the DAD. All variables were collected in the perioperative phase, defined as time from the start of anesthesia to discharge from the postoperative care unit.

Information on the choice of anesthetic agent was not available from the DAD.

### Statistical analysis

The study was performed as a survival study, using overall survival (OS) as the primary endpoint. OS was defined as the time from surgery to either death (all-cause mortality) or follow-up censoring. The latter was set to November 1st, 2021. Explanatory outcome variables are defined above. Explanatory variables were duration of surgery, perioperative blood transfusions, anesthesia technique and use of epidural analgesia.

Kaplan-Meier estimates were calculated for outcomes as a univariate interpretation of the individual perioperative factors. These results were compared by calculating log-rank tests for discrete variables. Hazard ratios (HR) were calculated using Cox-regression modelling in a univariate and a multivariate approach. The univariate approach associated OS with each of the explanatory variables.

The multivariate model was corrected for relevant confounders selected from domain knowledge of the current literature. We chose this approach to identify covariates as opposed to automated approaches such as stepwise regression, in order to avoid issues such as overfitting and inflated type-1 error rates associated with this approach [13].

These included demographics, perioperative and treatment related factors. Demographic covariates were Body Mass Index (BMI), sex, age, patient smoking status (non-smoker/active smoker or smoker within the last 8 weeks before surgery/unknown), alcohol use (non/1–21 drinks per week/more than 21 drinks per week/unknown) and comorbidities (Charlson Comorbidity Index, CCI) and American Society of Anesthesiology Score (ASA score). The CCI was calculated without age adjustment according to the original definition [14].

Perioperative covariates included operative time (minutes), epidural analgesia (yes/no), type of anesthesia (mixed intravenous and inhalation or TIVA), perioperative PRBC transfusions (yes/no). Information on critical bleeding (yes/no), as evaluated by the attending anesthesiologist, as well as pre-transfusion hemoglobin level was also extracted but not included as a covariate as this was seen as explanatory for transfusion rather than a unique covariate.

Treatment related factors included preoperative chemotherapy (yes/no), postoperative chemotherapy (yes/no), type of resection (PD/TP/DP). It should be noted that neoadjuvant chemotherapy was not used in Denmark for up-front resectable tumors during this study period, and all preoperative chemotherapy was thus given to downstage locally advanced or borderline tumors.

Tumor related factors included pathological tumor T-staging, pathology N-staging, and resection outcome (R0/R1/R2).

Data are presented as medians with interquartile range [IQR] or percentages, where appropriate. Kaplan-Meier survival estimates are plotted with the log-rank tests *p*-values. For group comparisons, the Mann-Whitney U-test was used to test differences between continuous variables whereas the chi-square test was used for the comparison of dichotomous variables.

The statistical R-suite was used for the analyses [15]. A *p*-value of < 0.05 was considered statistically significant.

### Missing data

Data were considered missing at random (MAR). Percentages of missing data are shown with the available data values where applicable. To assess the potential impact of missing data, an imputed data set was created using the predictive mean matching approach as implemented in the R “MICE” package. Supplementary Table 1 provides information on the results of the sensitivity analyses comparing the actual vs. the imputed dataset for the Cox regression results.

## Results

**Fig. 1:**
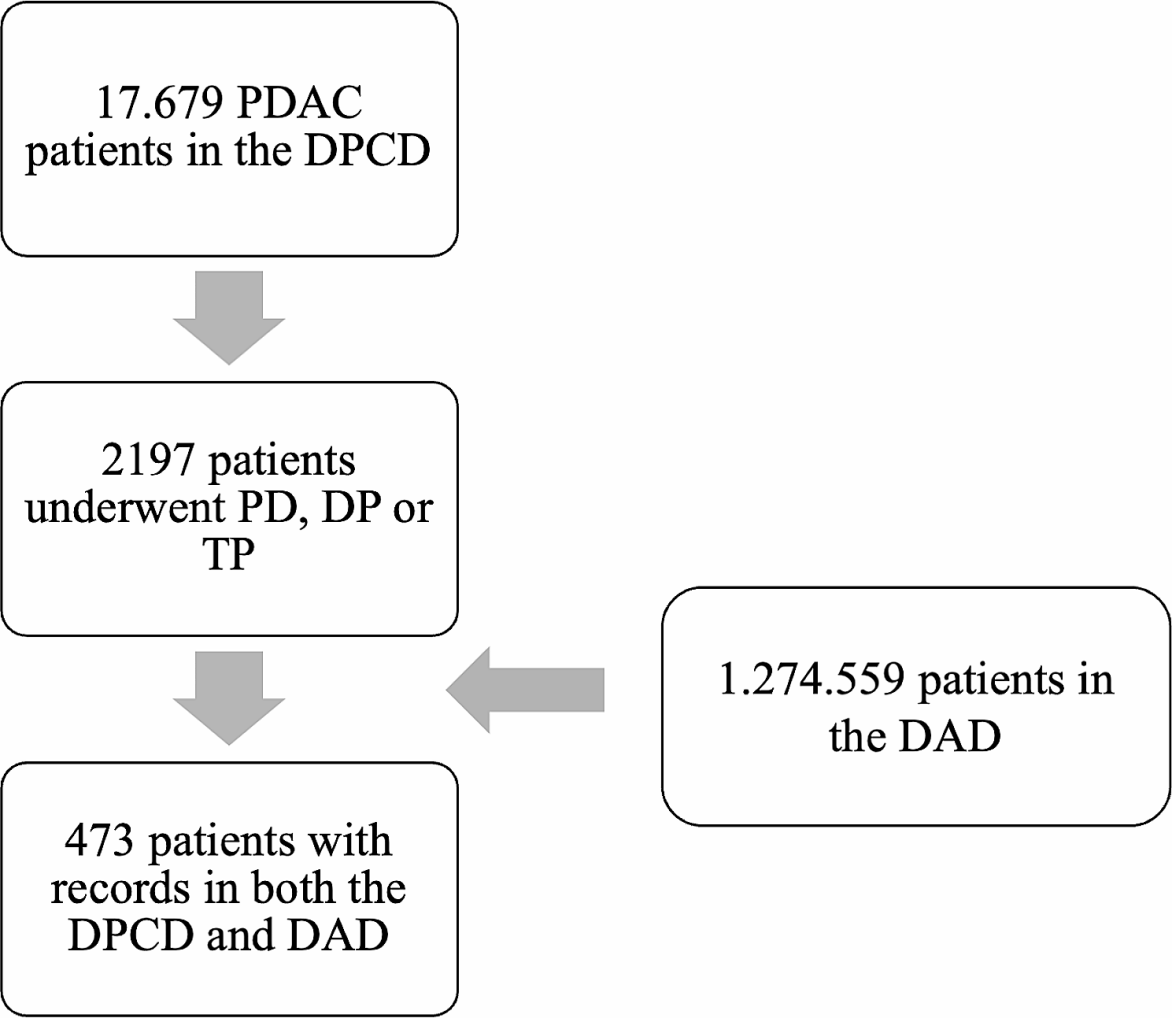
Overview of the patient inclusion flow. Patients having undergone pancreatic resections in the form of a pancreaticoduodenectomy (TP), distal pancreatectomy (DP) or total pancreatectomy (TP) for pancreatic adenocarcinoma from 2011 to 2020 at one of the four pancreatic surgery centers in Denmark were identified in the Danish Pancreatic Cancer Database (DPCD). This dataset was cross referenced with the Danish Anesthesia Database (DAD) for identification of perioperative data. In total, 473 patients had data available in both datasets, thus comprising the study population

Overall, perioperative (from the DAD) and PDAC related (from the DPCD) data were available from 473 patients from May 1st, 2011, to December 31st, 2020. Figure 1 shows a flowchart of the patient identification and inclusion.

Table 1 provides information on demographic, perioperative, treatment and tumor-related factors as well as overall outcomes for the cohort. Patients were predominantly male (54.9%), with a median age of 71[64–76]. A total of 55.7% of patients underwent pancreaticoduodenectomy, whereas 13.1% underwent total pancreatectomy and 16.3% distal pancreatectomy. Information on the specific procedure subtype was not available for the rest of the patients (14.9%). The median value of the American Society of Anesthesiology (ASA) score was 2 [2–3] and patients had a median Charlson Comorbidity Index of 1[0–2].

Nine transfused patients (10.7%) had pre-transfusion hemoglobin levels < 4.3 mmol/l, 59 (70.2%) had levels between 4.3 and 5.6 mmol/l and 16(19.0%) received transfusion with a pre-transfusion hemoglobin level > 5.6 mmol/l. Critical bleeding occurred in 33(8%) patients.

**Fig. 2:**
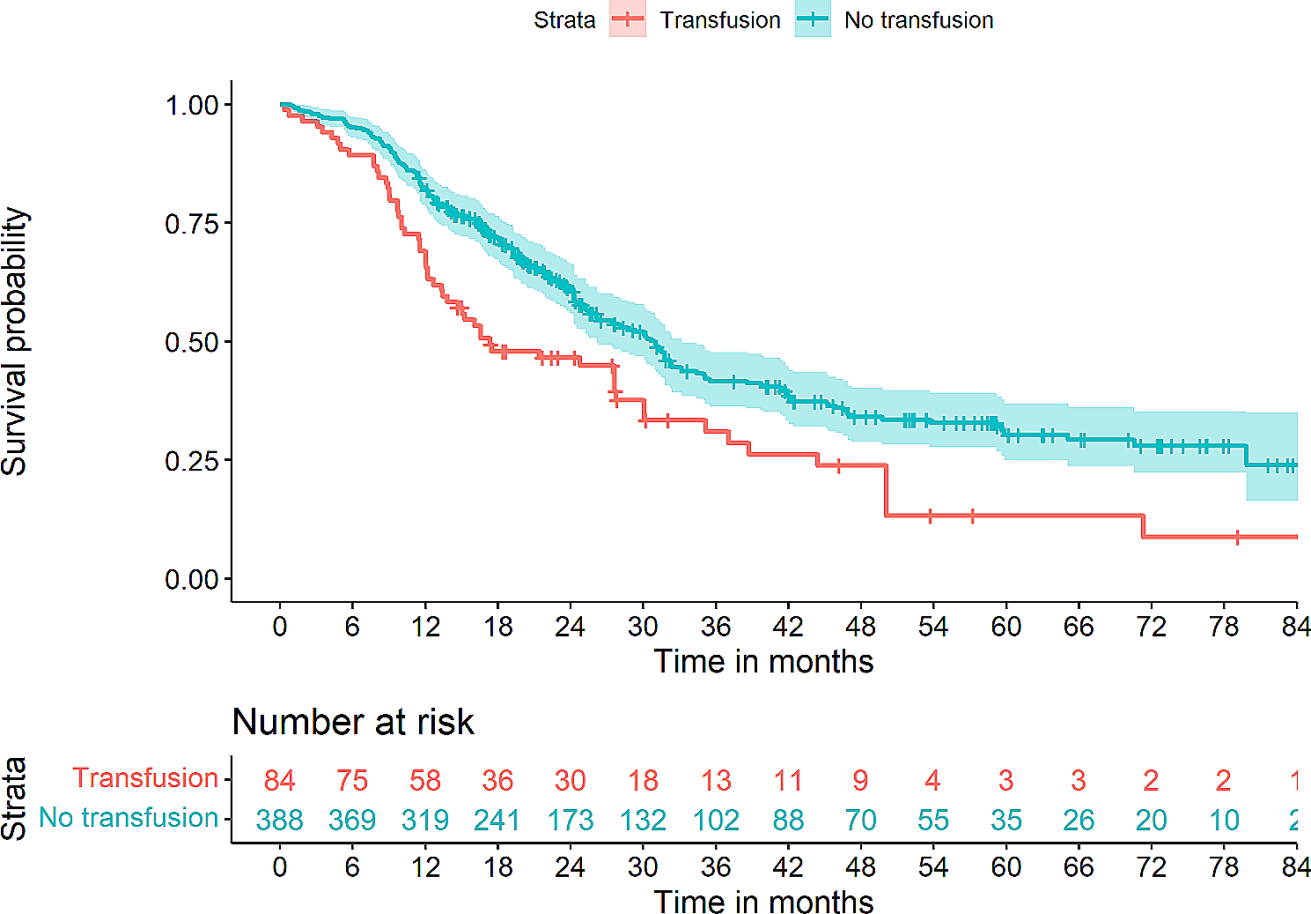
Kaplan-Meier curves depicting overall survival (OS) in patient strata either having received perioperative transfusions or no perioperative transfusions. Log-rank test *p* = 3 × 10^− 4^

**Fig. 3:**
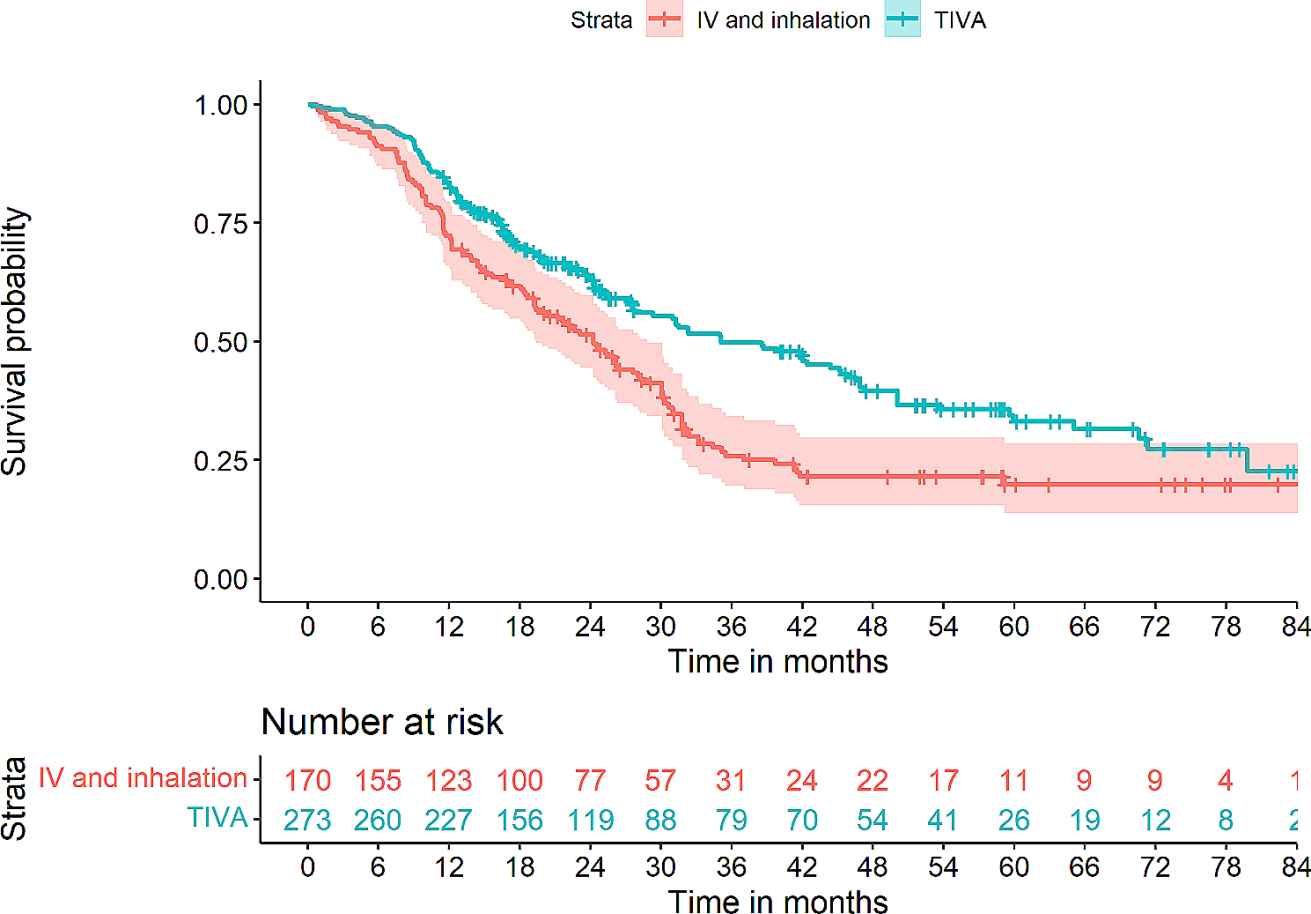
Kaplan-Meier curves depicting overall survival (OS) in patient strata either having received total intravenous anesthesia (TIVA) or mixed inhalation and intravenous anesthesia. Log-rank test *p* = 4 × 10^− 4^

**Fig. 4:**
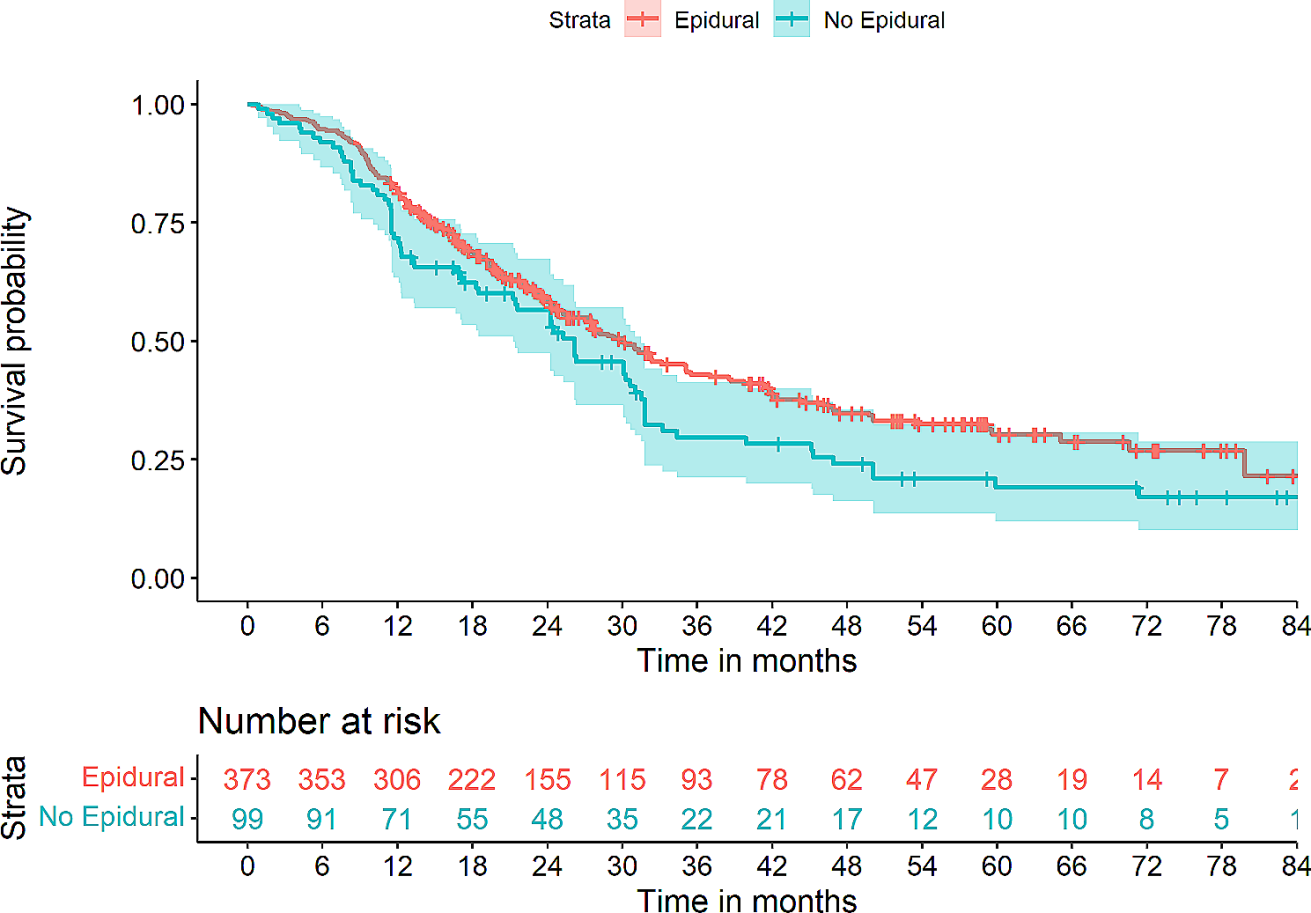
Kaplan-Meier curves depicting overall survival (OS) in patient strata either having received epidural analgesia or no epidural analgesia. Log rank test *p* = 0.06

Table 1 shows the estimated OS for discrete explanatory variables (blood transfusions, anesthesia technique and use of epidural analgesia) as well as a comparison of hospital length of stay (LOS) and progression to adjuvant chemotherapy when groups were stratified according to the analyzed explanatory variables (transfusion, analgesia, and type of anesthesia). Overall, no significant differences could be identified between groups in terms of LOS or transition to adjuvant chemotherapy. Figures 2, 3 and 4 provides a graphic overview of Kaplan-Meier curves for these variables.

**Table 1:** Demographic, treatment and tumor related variables from the *n* = 473 patients included in the study. Data are presented as medians [interquartile range] or percentages where appropriate

Category	Variable	Sub variable	Value
**Demographics**	Body Mass Index		25[22–27]
	Sex	Male (n, %)	259(54.9)
		Female (n,%)	213(45.1)
	Age (years)		71[64–76]
	Smoking	Non-smoker (n, %)	317(67.2)
		Active smoker* (n, %)	116(24.6)
		Unknown (n, %)	39(8.3)
	Alcohol use	Non (n, %)	106(22.5)
		1–21 drinks/week n, (n,%)	193(40.9)
		> 21 drink/week (n, %)	16(3.4)
		Unknown (n,%)	158(33.3)
	Charlson comorbidity index		1[0–2]
	ASA-score		2[2–3]
**Treatment related**	Hospital length of stay (days)		10[7–18]
	Duration of surgery (minutes)		278[236–323]
	Portal vein resection performed	Yes (n,%)	122(26.0)
		No (n,%)	351(74.0)
	Epidural analgesia	Yes (n,%)	373(79.0)
		No (n,%)	99(21.0)
	Type of anesthesia	Intravenous and inhalation (n,%)	170(36.0)
		Total Intravenous (n,%)	273(57.8)
		Unknown (n,%)	29(6.1)
	Perioperative transfusions	Yes (n, %)	84(17.8)
		No (n, %)	388(82.2)
	Pre-transfusion hemoglobin levels	>= 5.6 mmol/l (n,%)	16(19.0)
		>= 4.3 and < 5.6 mmol/l (n,%)	59(70.2)
		< 4.3 mmol/l (n,%)	9(10.7)
	Critical bleeding	Yes (n,%)	33(8.0)
		No (n,%)	440(93.0)
**Tumor related**	Preoperative chemotherapy	Yes (n,%)	43(9.1)
		No (n,%)	429(90.9)
	Postoperative chemotherapy	Yes (n,%)	370(78.4)
		No (n,%)	102(21.6)
	Type of resection	Pancreaticoduodenectomy (n,%)	263(55.7)
		Total pancreatectomy (n,%)	62(13.1)
		Distal pancreatectomy (n,%)	77(16.3)
		Unknown (n,%)	71(14.9)
	Tumor T-stage	T1(n,%)	30(6.4)
		T2(n,%)	86(18.2)
		T3(n,%)	243(51.5)
		T4(n,%)	9(1.9)
		Unknown (n,%)	104(22.0)
	Tumor N-stage	N0 (n,%)	104(22.0)
		N1 (n,%)	199(42.2)
		N2 (n,%)	59(12.5)
		Unknown (n,%)	110(23.3)
	Resection outcome	R0 (n,%)	211(44.7)
		R1 (n,%)	135(28.6)
		Unknown	126(26.7)
**Follow-up**	Status at end of follow-up	Alive (n,%)	196(41.5)
		Dead (n,%)	276(58.5)
	Follow-up time (months)		21[13–35]

Note: ASA: American Society of Anesthesiology Score.

*Defined as the time from the start of anesthesia to discharge from the postoperative care unit.

In the univariate Cox regression modelling (Table 2), shorter OS was associated with perioperative PRBC transfusions (HR 1.69, *p* = 3.55 × 10^− 4^), while longer OS was associated with epidural analgesia (HR 0.76, *p* = 0.045), combined inhalation/IV anesthesia (HR 0.69, *p* = 0.002) as well as a shorter procedure time (HR 0.99, *p* = 0.013). Significantly associated covariates included age (HR 1.02, *p* = 0.010), ASA score (HR 1.39, *p* = 0.004), alcohol use over 21 drinks per week (HR 0.73, *p* = 0.046), CCI (HR 1.18, *p* = 7.26 × 10^− 5^), postoperative chemotherapy (HR 0.58, *p* = 3.00 × 10^− 4^), tumour T3 (HR 4.08, *p* = 0.001) and N2 (HR 2.09, *p* = 0.004) staging as well as R1 resection margin (HR 0.014, *p* = 0.014) (Table 2).

**Table 2:** Overview of differences in estimated survival time, hospital length of stay (LOS) and progression to adjuvant chemotherapy for the studied outcomes. Hospital LOS is reported as medians [interquartile range, IQR]

	Perioperative transfusions		*p*-value	Anesthesia technique		*p*-value	Analgesia technique		*p*- value
Survival time* (months)	Yes	No		TIVA	IV + Inhalation		Epidural	No epidural	
**12**	66.7%	82.5%	NA	72.4%	82.8%	NA	81.8%	71.7%	NA
**24**	46.6%	60.9%	NA	51.5%	63.5%	NA	58.7%	56.5%	NA
**48**	23.9%	34.1%	NA	21.5%	39.6%	NA	34.9%	24.1%	NA
**72**	8.8%	28.0%	NA	19.9%	27.3%	NA	27.0%	17.0%	NA
**Hospital length of stay (days)**	11[6–20]	10[7–18]	0.512	10[7–19]	12[6–23]	0.917	10[7–18]	10[6–17]	0.818
**Progression to adjuvant chemotherapy (n,%)**	61(72.6)	309(65.3)	0.204	216(79.1)	154(90.5)	0.525	290(77.7)	80(80.8)	0.603

Note: * Kaplan-Meier estimates. Statistical analyses of differences in survival times were performed as Cox-regressions (see Tables 3 and 4).

In the multivariate model (Table 3), only PRBC transfusion retained the significant association with shorter OS (HR 2.53, *p* = 0.005). Covariates significantly associated with shorter OS in this model included CCI (HR 1.16, *p* = 0.028), ASA score (HR1.68, *p* = 0.039) and tumour N1 (HR = 2.23, *p* = 0.005) as well as N2 staging (HR 4.12, *p* = 0.002), whereas transition to postoperative chemotherapy was associated with longer OS (HR 0.53, *p* = 0.035). The results of the multivariate sensitivity analyses performed on the imputed dataset are shown in supplementary Table 1. Overall, the results were comparable to the multivariate results from the non-imputed dataset.

**Table 3:** Results of the univariate regression models

Category	Variable	Sub variable	Hazard Ratio	95% Confidence Interval	*p*-value
**Demographic**	Sex	Female	1.08	0.85–1.36	0.549
	Age		1.02	1.00-1.03	**0.010**
	ASA score		1.39	1.11–1.73	**0.004**
	Body mass index		0.98	0.95–1.01	0.254
	Charlson Comorbidity index		1.18	1.06–1.18	**7.26 × 10** ^**− 5**^
	Smoking	Current smoker	0.96	0.73–1.25	0.758
	Alcohol	1–21 drinks/week	0.72	0.35–1.48	0.369
		> 21 drinks/week	0.73	0.53–0.99	**0.046**
**Perioperative**	Procedure time		0.99	0.99–0.99	**0.013**
	Epidural analgesia		0.76	0.58–0.99	**0.045**
	Type of anesthesia§	Intravenous and inhalation	0.64	0.50–0.82	**0.002**
	Perioperative blood transfusion		1.69	0.45–0.79	**3.55 × 10** ^**− 4**^
**Tumor related**	Preoperative chemotherapy		0.88	0.57–1.35	0.564
	Postoperative chemotherapy		0.58	0.44–0.76	**3.00 × 10** ^**− 4**^
	Type of resection*	Total Pancreatectomy, TP	1.21	0.79–1.86	0.584
		Pancreaticoduodenectomy, PD	1.28	0.54–2.99	0.575
	Tumor T stage#	T2	4.23	1.66–10.78	**0.003**
		T3	4.08	1.97–11.70	**0.001**
		T4	3.11	0.90-10.75	0.083
	Tumor N stage**	N1	2.23	1.58–3.16	**6.11 × 10** ^**− 6**^
		N2	2.09	1.26–3.47	**0.004**
	Resection margin	R1	1.44	1.08–1.92	**0.014**

Note: §Total intravenous anesthesia (TIVA) used as reference.

* Distal Pancreatectomy used as reference.

#T1 tumor stage used as reference.

** N0 tumor stage used as reference.

**Table 4:** Results of the multivariate Cox-regression models

Category	Variable	Sub variable	Hazard Ratio	95% Confidence Interval	*p*-value
**Demographic**	Sex	Female	0.97	[0.59;1.60]	0.912
	Age		1.03	[1.00;1.06]	0.077
	ASA score		1.68	[1.03;2.74]	0.039
	Body mass index		0.97	[0.91;1.03]	0.357
	Charlson Comorbidity Index		1.16	[1.02;1.33]	**0.028**
	Smoking	Current smoker	1.33	[0.75;2.37]	0.332
	Alcohol	1–21 drinks/week	1.50	[0.46;4.94]	0.503
		> 21 drinks/week	2.33	[0.37;14.86]	0.369
**Perioperative**	Procedure time		1.00	[0.99;1.00]	0.540
	Epidural analgesia		1.39	[0.38;5.04]	0.619
	Type of anesthesia§	Intravenous and inhalation	0.70	[0.32;1.51]	0.365
	Perioperative blood transfusion		2.53	[1.32;4.87]	**0.005**
**Tumor related**	Preoperative chemotherapy		0.77	[0.25;2.35]	0.645
	Postoperative chemotherapy		0.53	[0.29;0.96]	**0.035**
	Type of resection*	Total Pancreatectomy, TP	1.69	[0.67;4.26]	0.269
		Pancreaticoduodenectomy, PD	0.79	[0.33;1.89]	0.593
	Tumor T stage#	T2	2.48	[0.51;12.09]	0.261
		T3	4.15	[0.89;19.32]	0.070
		T4	1.68	[0.18;15.72]	0.649
	Tumor N stage**	N1	2.76	[1.35;5.63]	**0.005**
		N2	4.12	[1.69;10.02]	**0.002**
	Resection margin	R1	1.10	[0.64;1.90]	0.737

Note: §Total intravenous anesthesia (TIVA) used as reference.

* Distal Pancreatectomy used as reference.

#T1 tumor stage used as reference.

** N0 tumor stage used as reference.

## Discussion

In this study we found a significant association between the use of perioperative PRBC transfusion and shorter OS after pancreatic resection for PDAC. In contrast, the use of epidural analgesia and the overall duration of surgery could not be associated with OS.

In this study, therefore, overall perioperative factors thus have limited impact on OS when adjusted for recognized covariates such as tumor pathology stages and the use of chemotherapy, although a potential negative effect of PRBC transfusions may exist.

Previous studies have associated duration of surgery with survival in PDAC surgery [8], an association that is also identified in the univariate analysis in this study. However, this association could not be confirmed using the multivariate approach where disease-centric factors such as tumor T and N classifications were accounted for. This suggests that the underlying severity of the disease may be the main driver of outcomes, and that duration of surgery serves as a surrogate for this rather than having an independent association with survival.

Multiple studies have identified associations between the immunological response to surgery, commonly referred to as the surgical stress response, and the risk of cancer recurrence in both general surgical oncology [16] as well as for pancreatic cancer surgery [17]. Overall, converging lines of evidence indicate that surgery induces profound immune suppression, including lymphopenia [18], dysfunction of natural killer (NK) cells [19], as well as dysregulation of the innate and adaptive immune responses as evidenced by T-cell suppression [20].

Furthermore, surgery induces a pro-metastatic environment through effects including direct tumor seeding as well as shielding of tumor cells from immune system surveillance through effects such as formation of neutrophil extracellular traps (NETs) [21] and platelet activation [21], essentially serving to encapsulate micrometastatic tumor cells and thus make these inaccessible for immune surveillance cells.

Blood transfusions have previously been shown to induce a pro-inflammatory response independently of other treatments (e.g. surgery) [22], and studies have also linked perioperative transfusions to an increased risk of cancer recurrence for patients after pancreatic cancer surgery [8, 23] as well as other solid tumors [24–26]. Interestingly, these effects appear to be independent of the amount of blood loss and preoperative anemia [24, 27]. Our results are consistent with these studies.

However, caution should be taken when interpreting these results. While the immune response promoted by blood transfusions may well be causally related to shorter OS through the mechanisms mentioned above, we cannot assess the indication, or the volume of transfusion deployed in this study. As such, transfusion triggers are unknown, and it could be speculated that transfused patients presented for surgery with lower hemoglobin levels due to more advanced or aggressive disease and that these factors rather than the blood transfusions per se were the reason for the shorter OS. Furthermore, transfusion strategies may be affected by the complexity of the procedure, especially in cases where the tumor is locally advanced, or portal vein resection is required. However, recent data from our group have indicated that portal vein resection itself is not associated with long-term survival differences [28]. Since preoperative chemotherapy was given only to downstage tumors and not for neo-adjuvant treatment of up-front resectable patients, this data point serves as a proxy for locally advanced tumors. As no association was identified between preoperative chemotherapy and OS in this study, locally advanced tumor status could not be associated with OS in this study. Furthermore, it should be noted that the transfusion rates presented here are lower than in many previously published studies, where rates of up to 45% of patients receiving blood transfusions have been reported [7, 29–31].

Regarding the anesthesia and analgesia strategies, converging lines of evidence also indicate that these factors may influence the surgical stress response. Studies have shown that the use of volatile inhalation anesthetics modulate the immune response to surgery through mechanisms including suppression of both the innate and adaptive immune responses [32], although reports are conflicting. Studies have indicated that volatile anesthetics may confer cytoprotective effects on cancer cells [33], results that are further supported by a meta-analysis from preclinical models collectively suggesting a pro-metastatic effect of volatile anesthetics [34] although high-quality clinical data supporting this hypothesis are currently lacking. In contrast, intravenous agents such as propofol may suppress the innate immune response and thereby attenuate the surgical stress response [35], although a recent review concluded that whether anesthetic techniques affect cancer recurrence still remain a controversial issues with evidence from large-scale randomized trials lacking [36]. Results from this study cannot support such an effect for PDAC surgery patients, although it should be emphasized that owing to the retrospective nature of this data set, factors such as differences in reporting practices and missing data do not allow us to assess the duration and volumes of anesthetic agents received.

Regarding analgesia techniques, the use of epidural analgesia has been suggested to attenuate the surgical stress response [37], although other studies have failed to support these findings [38]. Indeed, a recent randomized trial did not identify differences in recurrence rates between patients receiving regional or conventional analgesia for breast cancer surgery [39]. Although studies have suggested that opioids may worsen outcomes in the cancer setting [40], there are conflicting reports in the current literature on whether anesthesia and analgesia strategies affect OS following surgically oncological procedures. Our results do not support the notion that these factors have significant impact on OS following surgery for PDAC.

This study has limitations that should be recognized. First, as is the case with any retrospective study, results depend on the underlying data quality. Factors such as reporting practices, missing data and overall data quality may affect results. Although 2197 patients were identified in the DPCD, only 473 had matching records in the DAD. Several additional patients had records in both datasets with minor inconsistencies in terms of misalignment of procedure dates or subtypes, but we chose only to include patients with a complete match between databases in order not to distort the data. Potentially, this approach could introduce bias, as we cannot rule out that for instance more difficult cases were not recorded with as high a fidelity in both registries due to high clinical workloads. The presented results should therefore be assessed with this in mind.

Although we sought to identify and control for relevant covariates, other factors not accounted for in the multivariate analyses could potentially affect results. Specifically, it should be noted that data on postoperative complications are generally not available from the databases used, and these datapoints could thus not be included in the analyses. While complications are certainly relevant to surgical outcomes, recent unpublished work from our group suggests that major procedure-specific complications (e.g., anastomosis leakage) following PD after PDAC, may have limited impact on long-term survival and completion rates of chemotherapy. Thus, the absence of postoperative complication data points may have limited effects on the results presented in this study, although we cannot assess this through the available data in this study.

Second, it is important to recognize that treatment practices and outcomes may have changed during the study period, thus further affecting the presented results. Specifically, the transfusion rates reported here should be interpreted given that this study spans almost a decade. Transfusions practices have during this time moved from liberal to more restrictive, and the results presented should therefore be assessed bearing this in mind. This would in turn influence the overall fluid resuscitation strategies, which could also have impacted results.

The potential effect of confounding by indication should also be acknowledged, including cases where transfusions were administered because of low preoperative hemoglobin levels or epidural analgesia not used due to dysregulated coagulation status in the patient. In line with this, it should also be noted that outcomes of high-volume centers may be different from low volume centers. While all four centers included in this study are high-volume centers (> 20 procedures per year), the obvious difference in experience between a center performing 250 procedures and another performing 20 procedures may influence the results. The DPCD quality improvement program has, however, conducted national surveillance of the key performance quality indicators described in the Methods section over the past decade, and finding no significant differences between centers.

Third, owing to the retrospective nature of this study, we can only assess associations and not conclude on causality. Fourth, although the cohort is one of the largest PDAC datasets with perioperative data published to data, it still represents a national cohort of limited size. Differences in treatment practices between institutions may affect results, and results may not translate directly to other institutions internationally.

Even with these limitations, we conclude that a potential association between perioperative blood transfusion and reduced OS may exist for PDAC patients undergoing surgical resection, whereas we could not establish associations between anesthesia and analgesia strategies.

### Electronic supplementary material

Below is the link to the electronic supplementary material.

Supplementary Material 1

## Data Availability

Data are available upon reasonable request from the Danish Regions Quality Control Program (Regionernes Kliniske Kvalitetsskringsprogram, RKKP, www.rkkp.dk).

## References

[1] Wang H, Liu J, Xia G, Lei S, Huang X, Huang X (2020). Survival of pancreatic cancer patients is negatively correlated with age at diagnosis: a population-based retrospective study. Sci Rep.

[2] ECIS, European Cancer. Information System 2021 [Available from: https://ecis.jrc.ec.europa.eu.

[3] The global (2019). Regional, and national burden of pancreatic cancer and its attributable risk factors in 195 countries and territories, 1990–2017: a systematic analysis for the global burden of Disease Study 2017. Lancet Gastroenterol Hepatol.

[4] Lepage C, Capocaccia R, Hackl M, Lemmens V, Molina E, Pierannunzio D (2015). Survival in patients with primary liver cancer, gallbladder and extrahepatic biliary tract cancer and pancreatic cancer in Europe 1999–2007: results of EUROCARE-5. Eur J Cancer.

[5] Conroy T, Desseigne F, Ychou M, Bouché O, Guimbaud R, Bécouarn Y (2011). FOLFIRINOX versus gemcitabine for metastatic pancreatic cancer. N Engl J Med.

[6] Conroy T, Hammel P, Hebbar M, Ben Abdelghani M, Wei AC, Raoul JL (2018). FOLFIRINOX or Gemcitabine as Adjuvant Therapy for Pancreatic Cancer. N Engl J Med.

[7] Clark E, Connor S, Taylor MA, Hendry CL, Madhavan KK, Garden OJ (2007). Perioperative transfusion for pancreaticoduodenectomy and its impact on prognosis in resected pancreatic ductal adenocarcinoma. HPB (Oxford).

[8] Abe T, Amano H, Hanada K, Minami T, Yonehara S, Hattori M (2017). Perioperative Red Blood Cell Transfusion is Associated with Poor Long-Term Survival in pancreatic adenocarcinoma. Anticancer Res.

[9] Liu Z, Zhang J, Hong G, Quan J, Zhang L, Yu M (2016). Propofol inhibits growth and invasion of pancreatic cancer cells through regulation of the miR-21/Slug signaling pathway. Am J Transl Res.

[10] Chen W, Xu Y, Zhang Y, Lou W, Han X (2021). Positive Impact of Intraoperative Epidural Ropivacaine Infusion on oncologic outcomes in pancreatic Cancer patients undergoing pancreatectomy: a retrospective cohort study. J Cancer.

[11] Sugawara T, Ban D, Nishino J, Watanabe S, Maekawa A, Ishikawa Y (2021). Prediction of early recurrence of pancreatic ductal adenocarcinoma after resection. PLoS ONE.

[12] von Elm E, Altman DG, Egger M, Pocock SJ, Gotzsche PC, Vandenbroucke JP (2007). The strengthening the reporting of Observational studies in Epidemiology (STROBE) statement: guidelines for reporting observational studies. Lancet.

[13] Smith G (2018). Step away from stepwise. J Big Data.

[14] Charlson ME, Pompei P, Ales KL, MacKenzie CR (1987). A new method of classifying prognostic comorbidity in longitudinal studies: development and validation. J Chronic Dis.

[15] R Core Team. (2014). R: A language and environment for statistical computing. R Foundation for Statistical Computing, Vienna, Austria. URL http://www.R-project.org/.

[16] Chen Z, Zhang P, Xu Y, Yan J, Liu Z, Lau WB (2019). Surgical stress and cancer progression: the twisted tango. Mol Cancer.

[17] Romano F, Uggeri F, Crippa S, Di Stefano G, Scotti M, Scaini A (2004). Immunodeficiency in different histotypes of radically operable gastrointestinal cancers. J Exp Clin Cancer Res.

[18] d’Engremont C, Vernerey D, Pointet A-L, Simone G, Fein F, Heyd B (2016). Additive value of pre-operative and one-month post-operative lymphocyte count for death-risk stratification in patients with resectable pancreatic cancer: a multicentric study. BMC Cancer.

[19] Iannone F, Porzia A, Peruzzi G, Birarelli P, Milana B, Sacco L (2015). Effect of surgery on pancreatic tumor-dependent lymphocyte asset: modulation of natural killer cell frequency and cytotoxic function. Pancreas.

[20] Ananth AA, Tai LH, Lansdell C, Alkayyal AA, Baxter KE, Angka L (2016). Surgical stress abrogates pre-existing protective T cell mediated Anti-tumor Immunity leading to Postoperative Cancer recurrence. PLoS ONE.

[21] Alieva M, van Rheenen J, Broekman MLD (2018). Potential impact of invasive surgical procedures on primary tumor growth and metastasis. Clin Exp Metastasis.

[22] Suzuki G, Ichibayashi R, Masuyama Y, Yamamoto S, Serizawa H, Nakamichi Y (2022). Association of red blood cell and platelet transfusions with persistent inflammation, immunosuppression, and catabolism syndrome in critically ill patients. Sci Rep.

[23] Javed AA, Ronnekleiv-Kelly SM, Hasanain A, Pflüger MJ, Habib JR, Wright MJ et al. Blood transfusion is associated with worse outcomes following pancreatic resection for pancreatic adenocarcinoma. Annals Pancreat Cancer. 2022;5.

[24] Wu HL, Tai YH, Lin SP, Chan MY, Chen HH, Chang KY (2018). The impact of blood transfusion on recurrence and mortality following colorectal Cancer resection: a propensity score analysis of 4,030 patients. Sci Rep.

[25] Linder BJ, Frank I, Cheville JC, Tollefson MK, Thompson RH, Tarrell RF (2013). The impact of Perioperative Blood Transfusion on Cancer recurrence and survival following radical cystectomy. Eur Urol.

[26] Tartter PI. The Association of Perioperative Blood Transfusion with Colorectal Cancer recurrence. Ann Surg. 1992;216(6). 10.1097/00000658-199212000-00004 PMC1242709 1466616

[27] Pang QY, An R, Liu HL (2019). Perioperative transfusion and the prognosis of colorectal cancer surgery: a systematic review and meta-analysis. World J Surg Oncol.

[28] Sillesen M, Hansen CP, Dencker EE, Burgdorf SK, Krohn PS, Stender MT et al. Long-term outcomes of venous resections in pancreatic ductal adenocarcinoma patients: a Nationwide Cohort Study. Annals Surg Open. 2022;3(4). 10.1097/AS9.0000000000000219 PMC10406038 37600295

[29] Yeh JJ, Gonen M, Tomlinson JS, Idrees K, Brennan MF, Fong Y (2007). Effect of blood transfusion on outcome after pancreaticoduodenectomy for exocrine tumour of the pancreas. Br J Surg.

[30] Park HM, Park SJ, Shim JR, Lee EC, Lee SD, Han SS (2017). Perioperative transfusion in pancreatoduodenectomy: the double-edged sword of pancreatic surgeons. Med (Baltim).

[31] Sutton JM, Kooby DA, Wilson GC, Squires MH, Hanseman DJ, Maithel SK (2014). Perioperative Blood transfusion is Associated with decreased survival in patients undergoing pancreaticoduodenectomy for pancreatic adenocarcinoma: a multi-institutional study. J Gastrointest Surg.

[32] Stollings LM, Jia LJ, Tang P, Dou H, Lu B, Xu Y (2016). Immune Modulation Volatile Anesthetics Anesthesiology.

[33] Tavare AN, Perry NJ, Benzonana LL, Takata M, Ma D (2012). Cancer recurrence after surgery: direct and indirect effects of anesthetic agents. Int J Cancer.

[34] Hooijmans CR, Geessink FJ, Ritskes-Hoitinga M, Scheffer GJ (2016). A systematic review of the modifying effect of Anaesthetic Drugs on Metastasis in Animal models for Cancer. PLoS ONE.

[35] Kim R (2017). Anesthetic technique and cancer recurrence in oncologic surgery: unraveling the puzzle. Cancer Metastasis Rev.

[36] Ramirez MF, Cata JP. Anesthesia techniques and long-term oncological outcomes. Front Oncol. 2021;11. 10.3389/fonc.2021.788918 PMC8692375 34956903

[37] Wang J, Yin Y, Zhu Y, Xu P, Sun Z, Miao C (2019). Thoracic epidural anaesthesia and analgesia ameliorates surgery-induced stress response and postoperative pain in patients undergoing radical oesophagectomy. J Int Med Res.

[38] Kawasaki T, Ogata M, Kawasaki C, Okamoto K, Sata T (2007). Effects of epidural anaesthesia on surgical stress-induced immunosuppression during upper abdominal surgery. BJA: Br J Anaesth.

[39] Sessler DI, Pei L, Huang Y, Fleischmann E, Marhofer P, Kurz A (2019). Recurrence of breast cancer after regional or general anaesthesia: a randomised controlled trial. Lancet.

[40] Boland JW. Effect of opioids on survival in patients with Cancer. Cancers (Basel). 2022;14(22). 10.3390/cancers14225720 PMC9688536 36428812

