# Supplementary material for: Perioperative factors associated with survival following surgery for pancreatic cancer – a nationwide analysis of 473 cases from Denmark
Source: BMC Surg. 2024 Mar 2;24:76. doi: 10.1186/s12893-024-02369-4 (PMC10908011; doi:10.1186/s12893-024-02369-4)
Supplement: Supplementary file 1 — Supplementary Material 1 [file 12893_2024_2369_MOESM1_ESM.docx]

| **Category** | **Variable** | **Sub variable** | **Hazard Ratio** | **95% Confidence Interval** | **p-value** |
| --- | --- | --- | --- | --- | --- |
| **Demographic** | Sex | Female | 1.13 | 0.87-1.46 | 0.351 |
|  | Age |  | 1.01 | 0.99-1.02 | 0.287 |
|  | ASA score |  | 1.30 | 1.01-1.67 | **0.037** |
|  | Body mass index |  | 0.97 | 0.94-1.00 | 0.066 |
|  | Charlson Comorbidity index |  | 1.11 | 1.04-1.18 | **0.002** |
|  | Smoking | Current smoker | 0.86 | 0.65-1.16 | 0.325 |
|  | Alcohol | 1-21 drinks/week | 1.07 | 0.50-2.27 | 0.567 |
|  |  | >21 drinks/week | 0.71 | 0.50-1.03 | 0.069 |
|  |  |  |  |  |  |
| **Perioperative** | Procedure time |  | 0.99 | 0.99-1.01 | 0.337 |
|  | Epidural analgesia |  | 0.77 | 0.56-1.06 | 0.111 |
|  | Type of anesthesia§ | Intravenous and inhalation | 0.87 | 0.46-1.63 | 0.156 |
|  | Perioperative blood transfusion |  | 2.22 | 1.32-1.62 | **1.54x10^-6^** |
|  |  |  |  |  |  |
| **Tumor related** | Preoperative chemotherapy |  | 0.90 | 0.55-1.49 | 0.690 |
|  | Postoperative chemotherapy |  | 0.46 | 0.33-0.63 | **1.12x10^-6^** |
|  | Type of resection* | Total Pancreatectomy, TP | 0.75 | 0.53-1.08 | 0.856 |
|  |  | Pancreaticoduodenectomy, PD | 1.04 | 0.69-1.56 | 0.126 |
|  | Tumor T stage# | T2 | 2.92 | 1.34-6.37 | **0.007** |
|  |  | T3 | 4.41 | 2.13-9.14 | **6.44x10^-5^** |
|  |  | T4 | 2.72 | 0.98-7.52 | **0.054** |
|  | Tumor N stage** | N1 | 1.83 | 1.34-2.48 | **1.18x10^-4^** |
|  |  | N2 | 2.40 | 1.51-3.81 | **2.20x10^-4^** |
|  | Resection margin | R1 | 1.08 | 0.82-1.42 | 0.595 |

**SUPPLEMENTARY DATA**

**Supplementary table 1:** Results of the multivariate cox-regression models on the imputed dataset

§Total intravenous anesthesia (TIVA) used as reference

* Distal Pancreatectomy used as reference
#T1 tumor stage used as reference

** N0 tumor stage used as reference.
